# Proteomic Analysis Identifies Multiple Mechanisms of 5-Fluorouracil-Induced Gut Mucositis in Mice

**DOI:** 10.3390/cancers16234025

**Published:** 2024-11-30

**Authors:** Sergey M. Ivanov, Victor G. Zgoda, Valeria A. Isakova, Lyubov S. Trukhanova, Vladimir V. Poroikov, Alexander A. Shtil

**Affiliations:** 1 Institute of Biomedical Chemistry, 119121 Moscow, Russia; sergey.ivanov@ibmc.msk.ru (S.M.I.); vic@ibmc.msk.ru (V.G.Z.); vladimir.poroikov@ibmc.msk.ru (V.V.P.); 2 Department of Bioinformatics, Pirogov Russian National Research Medical University, 117513 Moscow, Russia; 3 Department of Biology, Saint-Petersburg University, 199034 Saint-Petersburg, Russia; isakova@scamt-itmo.ru; 4 Blokhin National Medical Research Center of Oncology, 115522 Moscow, Russia; m.yakubovskaya@ronc.ru; 5 Institute of Cyber Intelligence Systems, National Research Nuclear University MEPhI, 115409 Moscow, Russia

**Keywords:** 5-fluorouracil, toxicity, bioinformatics, proteomics, antitumor therapy

## Abstract

**Background/Objectives.** Damage of the gastrointestinal mucosa is a major side effect of the anticancer drug 5-fluorouracil (5-FU). Insight into the molecular pathogenesis of 5-FU-induced gut mucositis is expected to justify the strategies of prophylaxis. **Methods.** We analyzed intestinal specimens obtained from Balb/c mice treated with 70 mg/kg 5-FU daily for up to 6 days. **Results.** Manifestations of mucositis in the ileum and the colon included diarrhea, weight loss, and morphological lesions. The proteomic analysis revealed dozens of differentially expressed proteins governed by a set of master regulator proteins that regulated downstream pathways culminating in the complexes of specific transcription factors. Among the most important mechanisms of 5-FU-induced gut damage predicted by bioinformatics tools was stimulation of insulin-like growth factor 1 concomitant with inhibition of insulin receptor substrate 1, suggesting an involvement of the insulin pathway. Furthermore, the levels of 14-3-3γ protein and epinephrin B2 tyrosine kinase were interpreted as key inhibitory effects of 5-FU. These changes were detectable in the ileum as well as in the colon, pointing to the commonality of 5-FU responses across the gut. **Conclusion.** These results demonstrated a hierarchical network of gut injury mechanisms differentially regulated in the course of the emergence of 5-FU-induced mucositis.

## Simple Summary

One serious limitation of the efficacy of anticancer chemotherapy is the inevitable injury of non-malignant tissues. Side effects of 5-fluorouracil (5-FU), a drug widely used in treatment regimens for decades, are not an exception. Severe damage of the internal gut milieu, a state generally described by the term ‘mucositis’, is most unfavorable for the patient. This study presents an animal model for the analysis of molecular events attributable to the pathogenesis of 5-FU-induced gut mucositis. Our experiments and bioinformatics calculations indicated that this state is associated with a plethora of time-dependent changes in the intestine and the colon. These changes are directed at the coordination of the damage-related massive proteolysis combined with compensatory catabolic processes. Our findings uncovered new and counterintuitive mechanisms relevant to the rational design of pharmacological gut protectors during antitumor chemotherapy.

## 1. Introduction

Gastrointestinal cancer (GIC) remains a serious challenge for human health, being a major cause of cancer-related death worldwide [1,2]. Over the decades, 5-fluorouracil (5-FU) proved to be an efficient and virtually indispensable drug in many regimens of GIC chemotherapy [3,4]. However, side effects of 5-FU treatment, mainly, diffuse damage to the gut tissues, seriously hamper the therapeutic efficacy and quality of patients’ lives. This clinically unfavorable phenomenon is characterized by complex morphological and pathophysiological alterations that define gut mucositis. Generally, the effector mechanisms of this disorder are inflammatory cytokine bursts and the production of reactive oxygen species whose concerted action is critically important for mucosal damage. Apparently, these effector mechanisms are preceded by earlier changes; in turn, their manifold action would evoke a plethora of downstream events, making the manifestations of mucositis dependent on the dose and duration of drug exposure.

Initially, a variety of approaches to mitigate 5-FU-induced gut mucositis with natural agents have been explored, including probiotic-producing live microbial strains and plant extracts [5,6,7,8,9,10]. The discovery of molecular events triggered by 5-FU opened the opportunities for specific targeting. One unresolved issue on this route is whether a key regulator that governs the complex disorder can be identified and is this target druggable. A principally positive result has been demonstrated by He et al. [11]. In their experimental model, the 5-FU-induced intestinal injury was characterized by up-regulation of p53 and p53-regulated genes *p21* and *p16* and the formation of a senescence-like phenotype in mucosal cells. These changes were reversible with peficitinib, a Janus kinase inhibitor, indicating that targeting an individual regulatory pathway(s) can be efficient in the attenuation of mucositis. Importantly, peficitinib reinforced the antitumor efficacy of 5-FU, strongly supporting the therapeutic potential of this combination. Notwithstanding the pivotal significance of these findings, it remains to be proven whether the analyzed situation is not limited to a particular model. Indeed, the manifestations of mucositis, including the localization of lesions and the degree of tissue damage, are dependent on the regimen, that is, the dosage, the timing, and the route of administration. Moreover, the tissue alterations unfold over time so that the damage is accompanied by partial repair, further complicating the phenotype. Finally, the molecular mechanisms underlying mild vs. severe mucositis may differ. Thus, systemic studies are needed to dissect the multifactorial intestinal injury by 5-FU.

In this report, we investigated the proteome of the mouse intestine and colon in a model of severe mucositis induced by daily treatment with 5-FU for up to 6 days. We revealed robust alterations of epithelial morphology associated with multiple changes in tissue proteins. A bioinformatics analysis identified a variety of biochemical pathways and their master regulators, eventually culminating in gene transcription. These numerous alterations (1) comprise the complexity of the molecular pathogenesis of drug-induced mucositis and (2) provide opportunities for the prevention of gut injury via pharmacological modulation of individual mechanisms and/or their combinations.

## 2. Materials and Methods

### 2.1. Animals and Treatment

The rationale for 5-FU administration used in this study was the following. In our preliminary experiments, we aimed to establish a reproducible model of mucositis and avoid animal lethality. In so doing, we tested the range of 5-FU doses 50–100 mg/kg i.p. daily for up to 7 consecutive days. These treatments allowed us to achieve various degrees of symptom manifestations, from mild to severe. Commonly, our treatments were 60–70 mg/kg daily for 4–6 days. Lower doses caused less pronounced symptoms whereas doses >80 mg/kg were hardly tolerable. In the present study, we report 70 mg/kg daily as a reliable dose for single i.p. injection. As shown in Section 3.1, treatments for 6 days resulted in pronounced symptoms and robust tissue damage. By 4 days, the signs of gut mucositis were minimal, if any. Not before the model was validated by statistically convincing clinical manifestations and morphological picture did we start the proteomic analysis.

The Balb/c female mice (22–25 g, 12–14 weeks old) were propagated in the animal facility at Blokhin National Medical Research Center of Oncology. Mice were kept at 18–22 °C, 30–70% air humidity, and a 12 h light cycle. Animals received water and food ad libitum. Nine mice were divided into three groups, three animals per cohort. Mice in group 1 (mock-treated control) received i.p. injections of 0.3 mL saline for six consecutive days. Animals in the experimental cohorts were treated with 70 mg/kg 5-FU (Teva Pharmaceutical Industries, Tel-Aviv, Israel) i.p. in 0.3 mL saline daily for four (group 2) or six (group 3) days. Animals were monitored daily for general behavior, integrity of hair cover, weight loss, and stool. Diarrhea was categorized according to [12]: 1, normal; 2, mild (squishy stool); 3, moderate (unformed, somewhat watery stool); and 4, extreme diarrhea (watery stool and noticeable perianal stains). After the completion of treatments, mice were euthanized, and the ileum and the colon were excised and rinsed with saline. Two cm long full tissue pieces of the ileum (distal 2 cm from the stomach) and the colon (2.5 cm proximal distance from the anus) were cut and rinsed with saline. One-half of each tissue sample was immediately frozen in liquid nitrogen for proteomic analysis (see below). The second portion was fixed in 10% buffered formaldehyde. After fixation, the specimens were washed with water, dehydrated in alcohols–chloroform (70°–96°–100°), and placed into Histomix. Five µm tissue slices were cut, deparaffinated in xylol, treated with alcohols (100°–96°–70°), followed by rehydration, and stained with hematoxylin–eosin. Pieces of the ileum and the colon (>60 serial specimens per animal) were examined by light microscopy.

### 2.2. Mass Spectra (MS) Generation and Analysis

The tissue samples were lysed in 150 µL of the ice-cold buffer containing 5% sodium dodecyl sulfate and 100 mM triethylammonium bicarbonate, pH 7.5, and subjected to ultrasonication using the Bandelin Sonopuls probe (Bandelin Electronic GmbH and Co. KG, Berlin, Germany). Protein concentrations were measured using a Pierce™ BCA Protein Assay Kit (Pierce, Rockford, IL, USA). Then, trypsin digestion was performed according to the S-Trap sample preparation method (manufacturer’s manual available at https://protifi.com/, accessed on 3 March 2023).

The peptide samples were analyzed using UltiMate 3000 nano-flow HPLC system (Dionex, Sunnyvale, CA, USA) connected to Orbitrap Q-Exactive mass spectrometer (Thermo Fisher Sci., Waltham, MA, USA). Peptide separation was carried out on a Zorbax 300SB-C18 column, particle size 3.5 µm, 150 mm × 75 µm (Agilent Tech., Santa Clara, CA, USA), applying a linear gradient from 3% to 32% solvent B over 50 min, then from 32% to 53% solvent B over 3 min at 300 μL/min, followed by a washing step (5 min at 90% solvent B) and an equilibration step (5 min at 3% solvent B) at a flow rate of 0.3 µL/min.

MS were acquired in the positive ion mode with resolution of 70,000 (at *m*/*z* 400) for MS and 15,000 (*m*/*z* 400) for MS/MS scans. Survey MS scan was followed by MS2 spectra acquisition for the ten most abundant precursors. For peptide fragmentation, the higher energy collisional dissociation (HCD) was used, the signal threshold was set to 10,000 for an isolation window of 2 *m*/*z*, and the first mass of HCD spectra was set to 130 *m*/*z*. The normalized collision energy was set to 28. Singly charged ions and ions with no defined charge state were excluded from triggering MS/MS scans. Fragmented precursors were dynamically excluded from targeting for 10 s.

All MS2 spectra in the ‘raw’ format were processed using the Progenesis LC-MS software (version 4.1; Nonlinear Dynamics Ltd., Newcastle upon Tyne, UK). Protein identification was performed with Mascot software (version 2.4.1). The following search parameters were used: database, ‘SwissProt’ (version 2021_11) for the *Homo sapiens* species; the cutting enzyme, trypsin; peptide tolerance, 20 ppm; MS/MS-tolerance, ±0.05 Da. One possible missed trypsin cleavage site was allowed. Fixed modifications: cysteine carbamidomethyl; variable modifications, methionine oxidation. The criterion for positive identification was the score > 13; the significance threshold, *p* < 0.05; and FDR < 1%.

### 2.3. Pathway Enrichment Analysis and Functional Annotation of Proteins

Pathway enrichment analysis [13] allows identification of the pathways enriched with the studied proteins (genes) compared to the reference set of genes, e.g., all genes of the given organism in the Ensembl (https://www.ensembl.org) (accessed on 23 July 2023) database. The analysis was performed using g:Profiler (https://biit.cs.ut.ee/gprofiler/gost) (accessed on 23 July 2023) [14] service with default parameters. Pathways from KEGG (https://www.genome.jp/kegg/pathway.html) (accessed on 23 July 2023) and Reactome (https://reactome.org) (accessed on 23 July 2023), as well as Gene Ontology (GO; http://geneontology.org) (accessed on 23 July 2023) biological processes were taken into consideration. Since the GO data on pathways and processes did not cover all known functions of the identified proteins, we manually analyzed information about functions of individual proteins using UniProt database (https://www.uniprot.org) (accessed on 23 July 2023) and divided the proteins into functional groups. This procedure provided a detailed evaluation of protein functions.

### 2.4. Identification of Master Regulators and Potential 5-FU Targets

We postulated that 5-FU-induced changes in the abundance of gut proteins were preceded by changes in gene expression profile. Thus, we identified master regulators, i.e., proteins at the top of the signaling network that are responsible for induction and/or maintenance of gene expression. The analysis was performed using the Genome Enhancer (GE) platform (https://ge.genexplain.com) (accessed on 23 July 2023) [15]. The platform included three steps:

(i) Identification of promoters of differentially expressed genes. The promoter was defined as a region around the transcription start site from −1000 bp to +100 bp; total of 1100 bp.
(ii) Analysis of promoter regions to predict transcription factor (TF) binding sites (TFBSs) using positional weight matrices from the TRANSFAC database [16]. Since the combinations of TFs, not as much as a single TF, drive gene transcription and define its specificity, the combinations of TFBS sites termed ‘composite regulatory modules’ were identified [17]. To identify TFBSs and TFBS composite modules, the Match and the Composite Module Analyst (CMA) algorithms [18,19] were used. In these algorithms, the frequencies of TFBSs and TFBS composite modules in the promoters of studied genes were compared with the respective frequencies of the background set formed from the promoters of housekeeping genes. The TFs from the identified modules were used in the next step of analysis.
(iii) Reconstruction of the signaling network that activates the identified TFs to discover master regulators at the top of such a network. This analysis used the TRANSPATH database [20] and an algorithm based on the calculation of the shortest paths between the master regulator and TFs [21,22]. The analysis was performed separately for up- and down-regulated proteins in the ileum and the colon (four sets of proteins).

We selected two groups of master regulators for each protein set. First, we identified the master regulators differentially expressed in the gut of 5-FU-treated mice. This procedure was aimed at filtering out irrelevant factors that may not influence gene expression and/or may not even be expressed in the gut. Changes in transcription of genes encoding the selected master regulators mean that they are part of positive feedback loops and are critically important for the expression profiles.

Second, we selected the master regulators predicted as 5-FU targets using an analysis of structure–activity relationships (SARs). The prediction of activity spectra for substances (PASS) software (PASS 2022 Professional version) (http://www.way2drug.com/PASSOnline) analyzes a variety of biological activities including pharmacological effects, mechanisms of action (MOAs), toxic and adverse effects, interaction with metabolic enzymes and transporters, influence on gene expression, etc. [23]. The PASS procedure applies MNA (multilevel neighborhoods of atoms) descriptors and the modified naïve Bayes approach to analyze SARs based on a large training set of compounds. We applied the PASS Professional 2022 version to predict MOA based on the structural formula of 5-FU. MOA refers to a particular protein with information on the mode of functional changes: receptor agonist or antagonist, enzyme inhibitor/activator, etc. PASS calculates two values for each MOA: Pa is the probability that 5-FU exhibits a particular MOA, whereas Pi is the probability that 5-FU does not have the respective MOA. We selected the predicted 5-FU targets for further analysis based on the following criteria: (1) if the protein is a master regulator for up-regulated genes, and PASS predicts an increase in its function by 5-FU, i.e., 5-FU is an activator, this protein will be considered a potential 5-FU target; (2) if the protein is a master regulator for down-regulated genes, and PASS predicts its inhibition by 5-FU, i.e., 5-FU is an inhibitor, the protein will also be considered a 5-FU target.

## 3. Results

### 3.1. Validation of 5-FU-Induced Gut Mucositis in Mice

**Figure 1:**
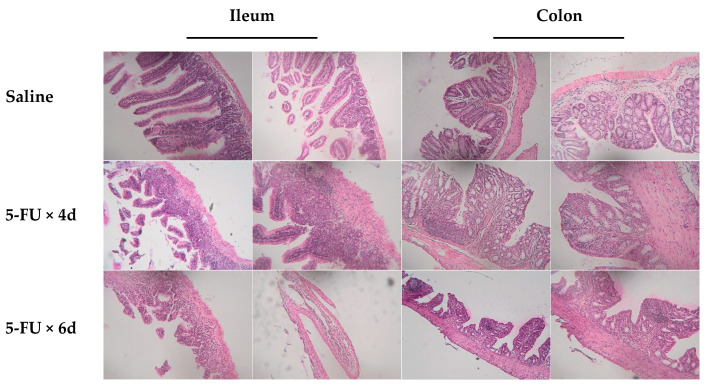
Histopathological signs of 5-FU-induced gut mucositis. Balb/c female mice were injected i.p. with saline (mock) or 70 mg/kg 5-FU for 4 or 6 days. Pieces of the ileum and colon were excised and processed for H and E staining. Shown are representative images selected from >60 view fields. Note the pronounced damage of the mucosa, that is, the loss of its integrity, injured villi, extensive vacuolization, and lymphocyte infiltration after 6 days of treatment. Initial magnification, 200×.

Commonly, the signs of experimental gut mucositis are detectable after i.p. injections of 50–100 mg/kg 5-FU for up to 7 days [6,24,25]. In our preliminary experiments, we tested this dose range and chose 70 mg/kg i.p. for 4–6 days as a tolerable regimen that yielded a reliable model of mucositis symptoms, allowing for monitoring animal behavior and gut morphology. Changes in hair cover, nutritional habits, behavioral reactions, or stool were not detectable in mice injected i.p. with 70 mg/kg 5-FU for 4 days (group 2, see Section 2.1). Mean weight loss was <10%. In contrast, a longer (6 days) treatment with 5-FU (group 3) led to a decreased activity of animals in the cage. Diarrhea was registered as scores 3 (two mice) and 4 (one animal); mean weight loss was 15–20%. In line with animal monitoring data, the histopathological examination of group 2 mice revealed no significant alterations in the structure of the ileum and colon compared to the mock-treated animals (Figure 1; top and middle panels). In striking contrast, in the group 3 animals, the morphological abnormalities characteristic of mucositis were robust (Figure 1; bottom panels; see also [6]). Thus, tissue specimens from groups 1 (control) and 3 (5-FUx6) were used for proteomic analysis.

### 3.2. Pathways Involved in 5-FU Responses in the Mouse Gut

**Figure 2:**
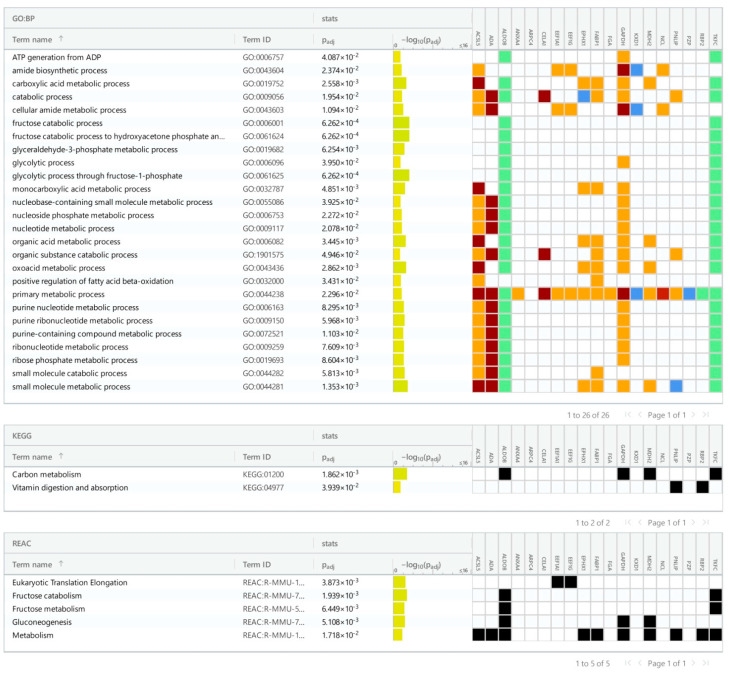
Pathways (KEGG and Reactome) and GO processes associated with up-regulated proteins in the colon of 5-FU-treated mice. The colors of cells represent the confidence of Gene Ontology term–gene associations.

**Figure 3:**
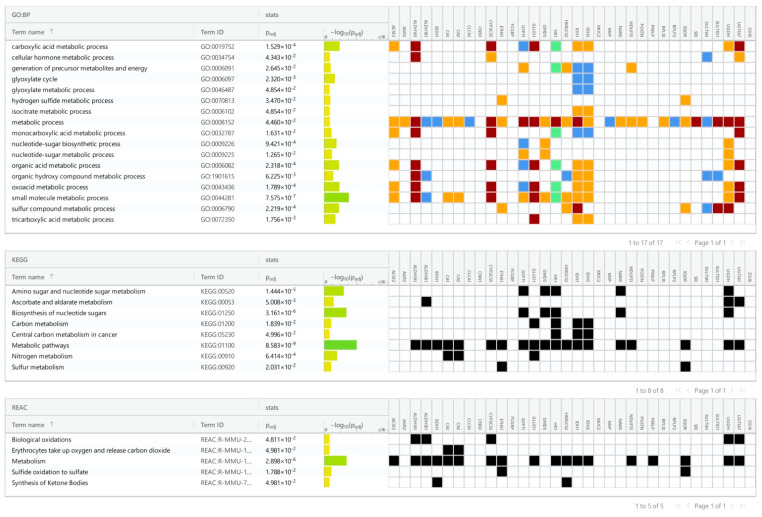
Pathways from KEGG and Reactome and GO processes associated with down-regulated proteins in the colon of 5-FU treated mice.

**Figure 4:**
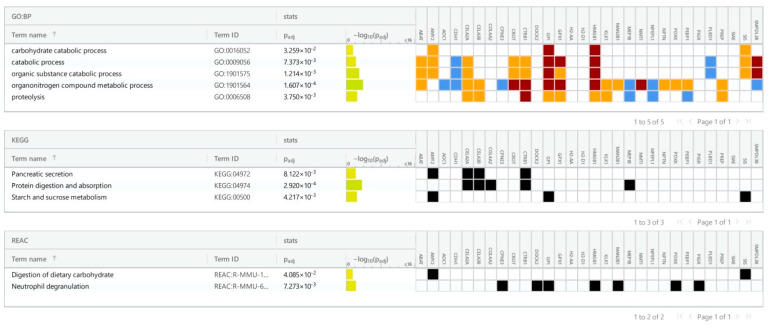
Pathways from KEGG and Reactome and GO processes associated with up-regulated proteins in the ileum of 5-FU treated mice.

**Figure 5:**
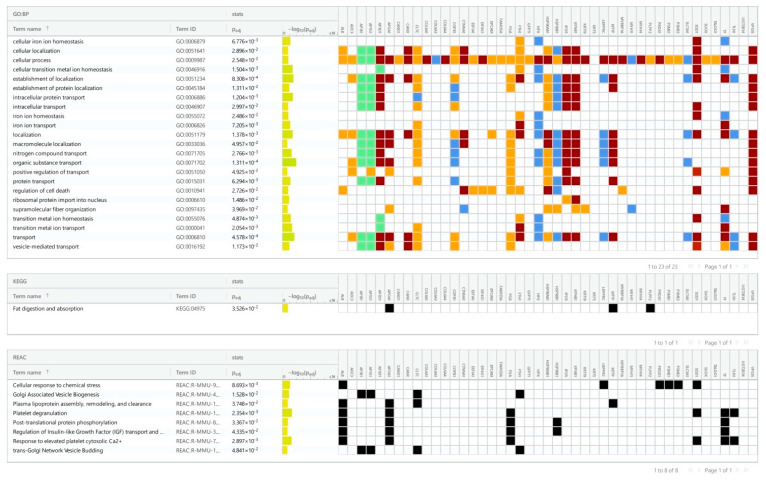
Pathways from KEGG and Reactome and GO processes associated with down-regulated proteins in the ileum of 5-FU treated mice.

We identified 32 up- and 46 down-regulated proteins in the ileum, as well as 19 up- and 34 down-regulated proteins in the colon. The pathway enrichment analysis was conducted separately for each of the four groups of differentially expressed proteins: up- and down-regulated proteins in each part of the gut (Figure 2, Figure 3, Figure 4 and Figure 5). In the colon, 5-FU activated catabolism of glucose, fructose, and fatty acids, the processes associated with ATP generation. In contrast, the metabolism of nucleotide sugars and other metabolic pathways was attenuated (Figure 2 and Figure 3).

Here and in Figure 3, Figure 4 and Figure 5, the colors represent the confidence of GO–gene associations. The highest confidence is marked red, followed by green, orange, and blue. The black color represents the protein in a particular KEGG or Reactome pathway.

As shown in Figure 4, 5-FU triggered proteolysis in the ileum. In particular, this treatment activated the proteases that hydrolyze the exogenous (i.e., diet) proteins. In contrast, down-regulated proteins were largely represented by transporters of peptides or ions (Figure 4).

### 3.3. Functional Groups of Differentially Expressed Proteins

**Figure 6:**
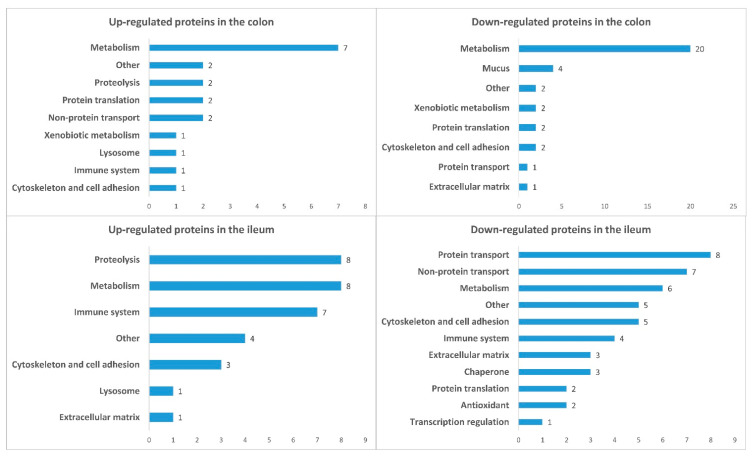
Functional groups of gut proteins differentially regulated in 5-FU-treated mice. The X-axis represents the number of proteins belonging to functional groups.

We used the description of gene function in the UniProt database to categorize the differentially expressed proteins. As shown in Figure 6, the majority of up- and down-regulated proteins in the colon were associated with metabolic pathways, whereas in the ileum the protein profiles were different. The Agr2, Clca1, Fcgbp, and Muc2 proteins involved in the colon mucosa formation were decreased. In contrast, proteins associated with proteolytic mechanisms were up-regulated in the ileum, while the proteins related to intracellular transport were decreased. Also, the proteins involved in the immune reactions were altered in the ileum (Figure 6 and Table 1 and Table 2).

### 3.4. Master Regulators of 5-FU-Induced Gene Expression in the Gut

Master regulators for up- and down-regulated genes in the gut of 5-FU-treated animals were identified using the GE platform (see Section 2). First, we determined the combinations of TFBSs called ‘composite regulatory modules’ that were preferentially presented on the promoters of studied genes compared with the housekeeping genes (control). These composite regulatory modules comprised the groups of TFs that were potentially responsible for the observed changes in gene and protein expression. Tentative TF complexes **1**,**2** were identified for each of the four cohorts (Table 3).

Next, we identified the master regulators upstream of TFs. These proteins were differentially regulated by 5-FU. One may hypothesize that up- or down-regulation of the individual master regulator would be translated into the subsequent change of mRNA abundance of the respective target gene. The selected master regulators are part of the positive feedback loops and are important for the induction and maintenance of transcription profiles (Table 4).

We predicted the protein targets of 5-FU along with MOAs using PASS 2022 Professional software (see Section 2). We intersected the predicted targets with master regulators on the basis of the following criteria: If the protein is a master regulator for up-regulated (or down-regulated) genes and if PASS predicts its stimulation (or inhibition) by 5-FU, this protein will be considered a regulator of 5-FU-induced gene/protein expression. Table 5 presents potential 5-FU targets as master regulators in the mouse ileum and colon. According to the results of PASS prediction, 5-FU may directly interact with these proteins and/or change their functions. This, in turn, would trigger alterations of signaling cascades and TF activities, culminating in changes in gene expression and protein abundance. The analysis identified the insulin-like growth factor 1 receptor (IGF1R) as the most probable 5-FU target. This protein was elevated in the ileum and in the colon. Other proteins commonly inhibited in both gut segments were Ca^2+^/calmodulin-dependent protein kinase A, the 14-3-3γ protein, ephrin B2 (EphB2) receptor tyrosine kinase, and the insulin receptor substrate 1 (IRS1). Two proteins reflected specific inhibitory effects of 5-FU in the ileum (S-100B) vs. colon (STAT1) (Table 5).

**Figure 7:**
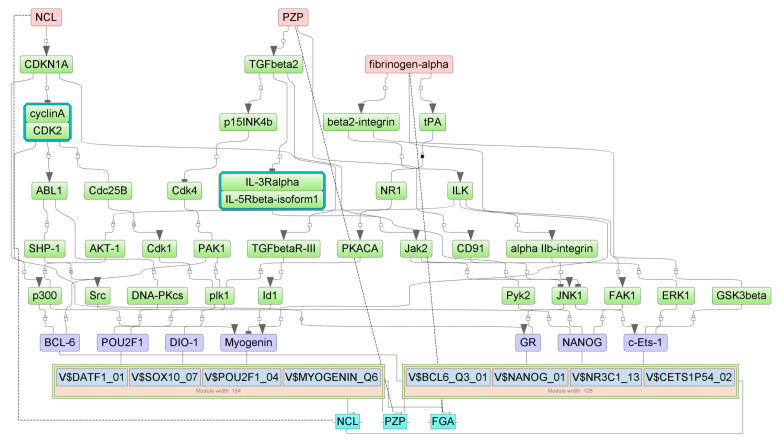
Pathways connecting 5-FU-induced genes with TFs, their potential complexes, and master regulators in the colon. Solid lines represent direct interactions between molecules, dashed lines represent gene expression.

**Figure 8:**
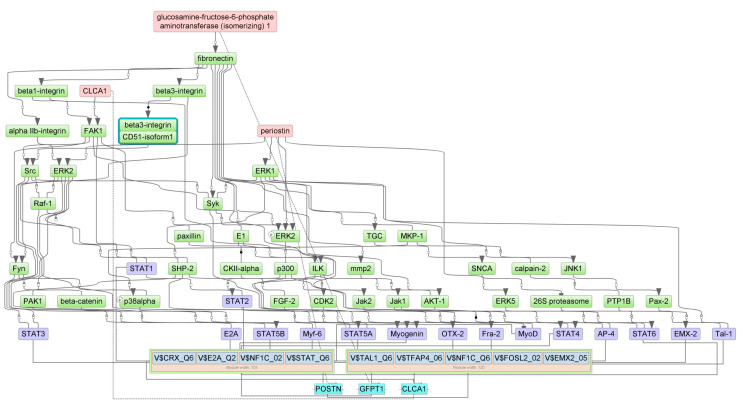
Pathways connecting 5-FU-attenuated genes with TFs, their potential complexes, and master regulators in the colon.

**Figure 9:**
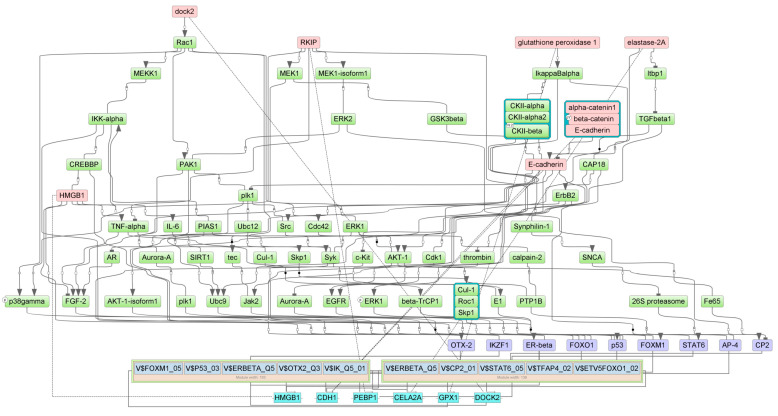
Pathways connecting 5-FU-induced genes with TFs, their potential complexes, and master regulators in the ileum.

**Figure 10:**
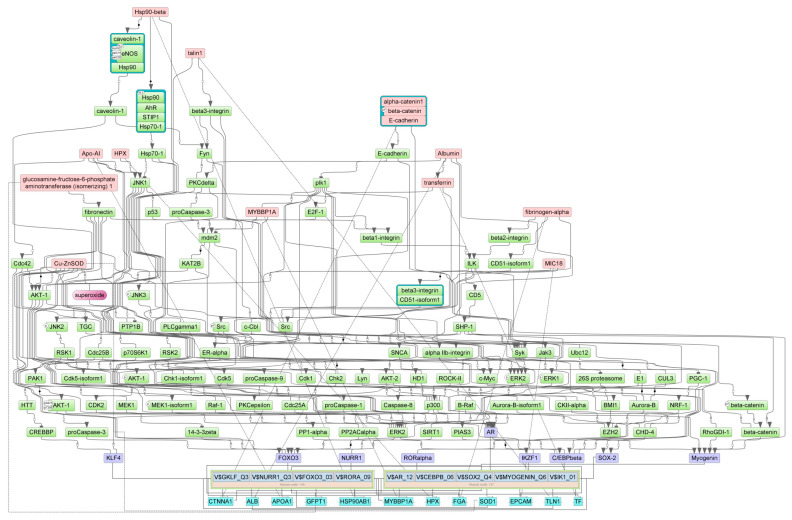
Interconnections between 5-FU-attenuated genes, TFs, their potential complexes, and master regulators in the ileum.

Figure 7, Figure 8, Figure 9 and Figure 10 show the signaling cascades that potentially regulate gene expression profiles in the gut of 5-FU-treated mice.

Here and in Figure 8, Figure 9 and Figure 10, master regulators are shown as pink rectangles. The composite regulatory modules are blue, and individual TFs are purple. Green rectangles are intermediate molecules added during the search for master regulators among the selected TFs. Cyan rectangles represent the genes that encode the master regulators.

## 4. Discussion

The proteomic data together with the bioinformatics analysis identified multiple levels of complexity of molecular changes in mice with 5-FU-induced acute gut mucositis. The observed changes included a variety of protein families such as ion transporters, metabolic enzymes, cytoskeletal and signaling proteins, transcriptional modulators, etc. These changes involved an overwhelmingly vast network of intermingled pathways, thereby forming the mechanistic basis for multiple and diverse manifestations of intestinal injury.

Most importantly, we found dramatic metabolic deregulation in the ileum and the colon, namely, the elevation of certain proteins concomitant with a drop of dozens of other markers. Bioinformatic tools used to obtain insight into the interconnections between the observed changes revealed a set of signaling pathways that culminated in individual TFs grouped in eight complexes characteristic for protein up- vs. down-regulation in the ileum and the colon (Table 3). These complexes termed master regulators comprised the effector mechanisms of differential gene deregulation in response to 5-FU. Interestingly, each complex contained a unique set of TFs suggesting a specific regulatory pattern for individual genes in the ileum and the colon. Although the majority of proteins changed to a relatively minor extent (~two-fold), we tend not to neglect their roles in the pathogenesis of 5-FU-induced mucositis.

In striking contrast to the diversity of TF complexes, the MOAs of 5-FU (interpreted as protein targets calculated by PASS algorithms) demonstrated a remarkable similarity in the two parts of the gut. In the ileum as well as in the colon, IGF1R activation emerged as the most probable MOA. Less probable but still notable was the concomitant inhibition of the protein substrate for this receptor, IRS1. Furthermore, the inhibition of Ca^2+^/calmodulin-dependent protein kinase A, the 14-3-3 family protein, and the receptor tyrosine kinase EphB2 were a shared MOA, whereas STAT1 decrease or S-100B activation were restricted to the colon and the ileum, respectively (Table 5). Such a commonality of MOA strongly suggests that the multiplicity of molecular effects of 5-FU converges to, or is governed by, a relatively narrow and specific set of mechanisms. Intriguingly, the list of these factors and their combinations were a priori unexpected.

The significance of insulin signaling in the regulation of stress responses is under extensive investigation. The tyrosine kinase activity of IGF1R has been attributed to cell protection. Stalnecker et al. performed a CRISPR/Cas9 loss-of-function screen and found that IGF1R was the top sensitizer of pancreatic cancer cells to the autophagy-inducing agent hydroxychloroquine [26]. Functionally, IGF1R synergized with the ERK-MAPR pathway in antagonizing autophagy. Furthermore, IGF1R countered the neurodevelopmental toxicity of decabromodiphenyl ether-209 via activation of downstream PI3K/AKT and ERK1/2 pathways [27]. The role of IRS1 in cell fate under cytotoxic stress is less understood. This protein has been shown to be decreased by doxorubicin or the marine carotenoid fucoxanthin in breast cancer cells [28]. IRS1 is known to interact with IGF1R given that the serine-rich region in the phosphorylation insulin resistance domain of IRS1 is not phosphorylated [29]. Multiple serine phosphorylation by different kinases abrogates IRS1 binding to IGF1R and interrupts downstream activation of pro-proliferative signaling leading to insulin resistance. In our experiments, 5-FU activated IGF1R and inhibited IRS1. In line with the report [26], we hypothesize that the former effect is compensatory, i.e., directed to spare the mucosa. However, IRS1 inhibition would disrupt the signaling thereby negating the tissue protective effect of IGF1R activation. It remains to be elucidated whether IGF1R activation is reciprocal with IRS1 inhibition. In other words, are these two processes regulated by 5-FU separately or in concert?

Next, PASS analysis identified the inhibition of Ca^2+^/calmodulin-dependent protein kinase A as a tentative MOA of 5-FU in the ileum and in the colon (Table 5). The significance of this mechanism in 5-FU-induced gut mucositis remains to be clarified. Aslam et al. [30] reported that 5-FU induced the Ca^2+^-dependent proteins including the cell membrane calcium-sensing receptor, calmodulin, and calmodulin-dependent kinase II in the mouse colon. However, the treatment with 5-FU (50 mg/kg/week for three consecutive weeks) was milder than the one used in the present study. The 5-FU dose dependence of Ca^2+^ responses is not surprising; however, whether inhibition of tissue Ca^2+^/calmodulin-dependent protein kinase A in the course of mucositis development is pathogenetically relevant or is just an epiphenomenon, deserves further investigation.

EphB receptor tyrosine kinases are markers of intestinal crypts near stem cell niches [31]. These kinases are involved in the structural organization of the epithelium via the formation of cobble-like cell morphology, polarization, patterning of the actin cytoskeleton, cell–cell communications, etc. [31]. Inhibition of EphB2 in the ileum and the colon (Table 5) may reflect the epithelial layer damage by 5-FU. Furthermore, this receptor is an upstream partner of Myc, and activation of the EphB2-Myc axis has been shown to precede the development of malignant transformation of the cultured esophageal epithelium [32]. Thus, on the one hand, EphB2 loss might be a factor of tissue damage that accompanies the severe mucositis (Figure 1); on the other hand, this MOA might be beneficial as an anticancer or a chemoprevention effect of 5-FU if EphB2 down-regulation holds true at lower drug doses.

The 14-3-3 family is comprised of proteins capable of forming homo- and/or heterodimer interactions along their N-terminal helices [see [33] for a recent review]. These proteins contain common modification domains, including the regions for divalent cation interaction and posttranslational modifications such as phosphorylation, acetylation, and proteolytic cleavage. Common recognition motifs of 14-3-3 proteins contain a phosphorylated serine or threonine residue. Mechanistically, the binding of 14-3-3 proteins to their peptide partners retains or sequesters signaling molecules in the cytoplasm. In the present study, we identified tyrosine 3-monooxygenase/tryptophan 5-monooxygenase activation protein γ (14-3-3γ), a family member encoded by the *YWHAG* gene, as a tentative MOA of 5-FU in the mouse ileum and the colon. This highly conserved protein is a part of cellular stress responses in plants and mammals [34,35]. Furthermore, 14-3-3γ is inducible by growth factors in human vascular smooth muscle cells and is highly expressed in skeletal and heart muscles, suggesting an important role of 14-3-3γ in the muscle tissue. This protein has been shown to interact with Raf1 and protein kinase C, the key signaling proteins in various pathways. Several studies strongly suggested that 14-3-3γ is an oncogene ([36] and refs therein). Recently, *YWHAG*/14-3-3γ was found among the prognosis-relevant DNA damage and repair signature genes in glioma cells [35]. Interestingly, 14-3-3σ has been attributed to a positive regulation of IGF1R signaling [37]. One may hypothesize that IGF1R, IRS1, and 14-3-3γ comprise a signaling axis targeted by 5-FU.

Recent reports indicated a series of natural compounds capable of preventing 5FU-induced tissue injury [24,38,39,40,41]. Nevertheless, the robust multiplicity and versatility of 5-FU effects in the gut demonstrated in the present study comprise the complexity of the molecular pathogenesis of mucositis. Apparently, it is hardly possible to specify one essential mechanism and one single target for pharmacological prevention of this unfavorable side effect of the otherwise important drug. Investigation into early events that precede the overt manifestations of mucositis, in particular, the genome-wide profiling of rapid expression in single cells of the gut mucosa, is expected to identify the primary upstream master regulators as druggable targets.

## 5. Limitation of This Study

The proteomic analysis was performed at a one-time point to specifically characterize the manifested mucositis. Nevertheless, this study identified a set of alterations that may serve as key markers of the unveiling of tissue injury. Time course and dose-response experiments are definitely needed. One may expect that milder or more severe regimens of 5-FU administration would be associated with differential patterns of mRNA and protein levels. At the present stage of our investigation, we aimed at the initial characterization of protein imbalance in the ileum vs. colon associated with advanced mucositis. It is interesting to track the earliest changes of key protein markers that can occur prior to pathophysiological and morphological manifestations of gut injury. However, the omics experiments and bioinformatics analyses are laborious and require big animal cohorts; the latter issue poses certain ethical concerns. Now, with the primary mechanistic knowledge, the gut damage by 5-FU should be examined in detail. In particular, the comprehensiveness of the information provided in the present study would be further substantiated by microdissection of gut samples and determining cell type-specific effects of 5-FU in individual cell types by single-cell RNA sequencing.

## 6. Conclusions

This study presents a generalized picture of multiple changes in gene and protein expression in the mouse gut tissues damaged by 5-FU. The observed findings comprise an exceptionally complex and multidirectional mechanism of local side effects of the clinically important drug. Principal problem raised by our analysis is whether there is one common mechanism that governs all downstream events. If the answer is positive, such mechanism can be a candidate for therapeutic targeting. However, the diversity of molecular changes makes this assumption doubtful. Consequently, it is premature to build a strategy of prophylaxis of 5-FU-induced gut mucositis based on single targeting. On the other hand, the identified pathways, although manifold, are subject to an individual hierarchy; hopefully, pharmacological and/or genetic modulations of particular master regulators represent a perspective approach.

## Figures and Tables

**Table 1:** Differentially expressed proteins associated with metabolic pathways.

Protein Name	UniProtID	Gene Symbol	Fold Change *
**Proteins up-regulated in the colon**			
Adenosine deaminase	P03958	Ada	3.2
Bifunctional ATP-dependent dihydroxyacetone kinase/FAD-AMP lyase (cyclizing)	Q8VC30	Tkfc	3.8
Fructose-bisphosphate aldolase B	Q91Y97	Aldob	4.5
Glyceraldehyde-3-phosphate dehydrogenase	P16858	Gapdh	2.3
Long-chain-fatty-acid–CoA ligase 5	Q8JZR0	Acsl5	2.9
Malate dehydrogenase, mitochondrial	P08249	Mdh2	2.4
Pancreatic triacylglycerol lipase	Q6P8U6	Pnlip	11.9
**Proteins down-regulated in the colon**			
Acyl-CoA synthetase family member 2, mitochondrial	Q8VCW8	Acsf2	−14.2
Aldehyde dehydrogenase X, mitochondrial	Q9CZS1	Aldh1b1	−5.5
Carbonic anhydrase 1	P13634	Ca1	−38.4
Carbonic anhydrase 2	P00920	Ca2	−7.1
Cytochrome P450 2C55	Q9D816	Cyp2c55	−7.5
D-beta-hydroxybutyrate dehydrogenase, mitochondrial	Q80XN0	Bdh1	−5.6
GDP-mannose 4,6 dehydratase	Q8K0C9	Gmds	−2.2
Glutamate dehydrogenase 1, mitochondrial	P26443	Glud1	−2.0
**Glutamine--fructose-6-phosphate aminotransferase [isomerizing] 1**	P47856	Gfpt1	−5.9
Hexokinase-1	P17710	Hk1	−2.8
Hydroxymethylglutaryl-CoA synthase, mitochondrial	P54869	Hmgcs2	−8.6
Isocitrate dehydrogenase [NADP] cytoplasmic	O88844	Idh1	−2.5
Isocitrate dehydrogenase [NADP], mitochondrial	P54071	Idh2	−2.6
N-acetylneuraminic acid synthase (sialic acid synthase)	Q99J77	Nans	−4.2
NADH-ubiquinone oxidoreductase 75 kDa subunit, mitochondrial	Q91VD9	Ndufs1	−2.3
Protein ETHE1, mitochondrial	Q9DCM0	Ethe1	−6.0
Retinal dehydrogenase 1	P24549	Aldh1a1	−2.2
Sulfide:quinone oxidoreductase, mitochondrial	Q9R112	Sqor	−5.2
UDP-glucose 6-dehydrogenase	O70475	Ugdh	−12.5
UDP-glucuronosyltransferase 1-7C	Q6ZQM8	Ugt1a7	−2.4
**Proteins up-regulated in the ileum**			
4-aminobutyrate aminotransferase, mitochondrial	P61922	Abat	4.7
Amiloride-sensitive amine oxidase [copper-containing]	Q8JZQ5	Aoc1	7.8
Glucose-6-phosphate isomerase	P06745	Gpi	12.0
Glutathione peroxidase 1	P11352	Gpx1	4.1
Pancreatic alpha-amylase	P00688	Amy2	2.5
Peroxisomal carnitine O-octanoyltransferase	Q9DC50	Crot	4.2
Protein Sis	-	Sis	2.7
Pyridoxal kinase	Q8K183	Pdxk	2.5
**Proteins down-regulated in the ileum**			
**Glutamine--fructose-6-phosphate aminotransferase [isomerizing] 1**	P47856	Gfpt1	−7.5
Lipid phosphate phosphohydrolase 2	Q9DAX2	Plpp2	−2.1
Membrane primary amine oxidase	O70423	Aoc3	−8.9
Protein Ugt2b34	-	Ugt2b34	−2.2
Sulfite oxidase, mitochondrial	Q8R086	Suox	−2.4
Transaldolase	Q93092	Taldo1	−2.2

Fold changes of up-regulated and down-regulated proteins are shown in red and blue, respectively. The underlined bold means that the protein was differentially expressed in the colon and the ileum. * Negative fold change value means that the protein abundance was decreased by 5-FU treatment.

**Table 2:** Differentially expressed proteins not associated with metabolic pathways.

Functional Group	Protein Name	UniProtID	Gene Symbol	Fold Change *
**Proteins up-regulated in the colon**				
Cytoskeleton and cell adhesion (actin polymerization, DNA repair)	Actin-related protein 2/3 complex subunit 4	P59999	Arpc4	2.0
Immune system (blood coagulation, innate and adaptive immunity)	**Protein Fga**	E9PV24	Fga	2.4
Lysosome (regulation of lysosome size and localization)	KxDL motif-containing protein 1 (fragment)	Q80XH1	Kxd1	2.3
Non-protein transport (transport of lipids)	Fatty acid-binding protein, liver	P12710	Fabp1	8.9
Non-protein transport (transport of retinoid)	Retinol-binding protein 2	Q08652	Rbp2	2.9
Other	Annexin A4	P97429	Anxa4	2.3
Other	Nucleolin OS	P09405	Ncl	3.4
Protein translation factor	Elongation factor 1γ	Q9D8N0	Eef1g	2.3
Protein translation factor	**Elongation factor 1α-1**	P10126	Eef1a1	2.1
Proteolysis (digestive enzyme)	Chymotrypsin-like elastase family member 1	Q91 × 79	Cela1	22.2
Proteolysis (protease inhibitor)	Protein Pzp	Q61838	Pzp	2.1
Xenobiotic metabolism	Epoxide hydrolase 1	Q9D379	Ephx1	5.8
**Proteins down-regulated in the colon**				
Cytoskeleton and cell adhesion (cell adhesion)	Periostin	Q62009	Postn	−3.1
Cytoskeleton and cell adhesion (regulation of muscle contraction)	Calponin-1	Q08091	Cnn1	−4.2
Extracellular matrix	Prolargin	Q9JK53	Prelp	−4.8
Mucus (maintenance of the mucosal structure)	Protein Fcgbp	-	Fcgbp	−20.6
Mucus (major constituent of mucus)	Mucin-2 (Fragments)	Q80Z19	Muc2	−3.6
Mucus (regulation of mucus production)	Calcium-activated chloride channel regulator 1	Q9D7Z6	Clca1	−12.5
Mucus (required for mucus synthesis and secretion)	Anterior gradient protein 2 homolog	O88312	Agr2	−6.0
Other	Major vault protein	Q9EQK5	Mvp	−2.4
Other	Sorcin	Q6P069	Sri	−3.4
Protein translation (ribosomal protein)	60S acidic ribosomal protein P2	P99027	Rplp2	−2.1
Protein translation (ribosomal protein)	60S ribosomal protein L10	Q6ZWV3	Rpl10	−2.1
Protein transport	Zymogen granule membrane protein 16	Q8K0C5	Zg16	−20.1
Xenobiotic metabolism	Sulfotransferase 1 family member D1	Q3UZZ6	Sult1d1	−14.9
Xenobiotic metabolism	Sulfotransferase 1A1	P52840	Sult1a1	−20.2
**Proteins up-regulated in the ileum**				
Cytoskeleton and cell adhesion (cell–cell adhesions)	Cadherin-1	P09803	Cdh1	2.4
Cytoskeleton and cell adhesion (cell–cell adhesions)	Neuroplastin (fragment)	P97300	Nptn	2.5
Cytoskeleton and cell adhesion (cytoskeleton regulation)	Dedicator of cytokinesis protein 2	Q8C3J5	Dock2	3.6
Extracellular matrix (structural protein)	Collagen α-2(IV) chain	P08122	Col4a2	2.8
Immune system	High mobility group protein B1	P63158	Hmgb1	2.1
Immune system (immunoglobulin binding)	Polymeric immunoglobulin receptor	O70570	Pigr	6.0
Immune system (lipid composition and membrane fluidity)	Acid sphingomyelinase-like phosphodiesterase 3b	P58242	Smpdl3b	2.4
Immune system (MHC subunit)	H-2 class I histocompatibility antigen, D-D α chain	P01900	H2-D1	2.5
Immune system (MHC subunit)	H-2 class II histocompatibility antigen, A-D α chain	P04228	H2-Aa	2.5
Immune system (MHC subunit)	H-2 class II histocompatibility antigen, E-D β chain	P01915	-	3.9
Immune system (MHC subunit)	H-2 class II histocompatibility antigen, E-K α chain	P04224	-	3.5
Lysosome (enzyme)	Lysosomal α-mannosidase	O09159	Man2b1	2.2
Other	Copine-3	Q8BT60	Cpne3	2.4
Other	Glycylpeptide N-tetradecanoyltransferase 1	O70310	Nmt1	3.2
Other	Phospholipase B-like 1	Q8VCI0	Plbd1	2.2
Other	Sialate *O*-acetylesterase	P70665	Siae	2.7
Proteolysis	Isoform 2 of meprin A subunit β	Q61847	Mep1b	2.5
Proteolysis	Kallikrein-1	P15947	Klk1	2.4
Proteolysis	Probable aminopeptidase NPEPL1	Q6NSR8	Npepl1	2.1
Proteolysis	Prolyl endopeptidase	Q9QUR6	Prep	4.8
Proteolysis (digestive enzyme)	Chymotrypsin-like elastase family member 2A	P05208	Cela2a	4.7
Proteolysis (digestive enzyme)	Chymotrypsin-like elastase family member 3B	Q9CQ52	Cela3b	3.8
Proteolysis (digestive enzyme)	Chymotrypsinogen B	Q9CR35	Ctrb1	2.6
Proteolysis (protease inhibitor)	Phosphatidylethanolamine-binding protein 1	P70296	Pebp1	2.2
**Proteins down-regulated in the ileum**				
Antioxidant	Peroxiredoxin-1 (fragment)	P35700	Prdx1	−4.5
Antioxidant	Superoxide dismutase [Cu-Zn]	P08228	Sod1	−2.0
Chaperone	Calnexin	P35564	Canx	−2.2
Chaperone	Endoplasmin OS	P08113	Hsp90b1	−6.2
Chaperone	Heat shock protein HSP 90β	P11499	Hsp90ab1	−4.1
Cytoskeleton and cell adhesion (cell–cell adhesions)	Catenin α-1	P26231	Ctnna1	−27.3
Cytoskeleton and cell adhesion (cell–cell adhesions)	Epithelial cell adhesion molecule	Q99JW5	Epcam	−4.2
Cytoskeleton and cell adhesion (cytoskeleton-membrane connection and cell–cell adhesion)	Talin-1	P26039	Tln1	−3.8
Cytoskeleton and cell adhesion (muscle contraction)	Myosin-11	O08638	Myh11	−2.7
Cytoskeleton and cell adhesion (muscle contraction)	Myosin-14	Q6URW6	Myh14	−2.5
Extracellular matrix (structural protein)	Collagen α-1(VI) chain	Q04857	Col6a1	−2.1
Extracellular matrix (structural protein)	Collagen α-4(VI) chain	A2AX52	Col6a4	−2.6
Extracellular matrix (structural protein)	Protein Col6α3	-	Col6a3	−2.4
Immune system (blood coagulation, innate and adaptive immunity)	**Protein Fga**	E9PV24	Fga	−9.8
Immune system (immunoglobulin)	Ig-γ-2A chain C region, A allele	P01863	Ighg	−2.9
Immune system (immunoproteasome component)	Proteasome activator complex subunit 1 (fragment)	P97371	Psme1	−2.3
Immune system (immunoproteasome component)	Proteasome activator complex subunit 2	P97372	Psme2	−2.8
Non-protein transport	Serum albumin	P07724	Alb	−5.9
Non-protein transport (transport of glucose)	Sodium/glucose cotransporter 1	Q8C3K6	Slc5a1	−21.1
Non-protein transport (transport of iron)	Ferritin heavy chain	P09528	Fth1	−3.7
Non-protein transport (transport of iron)	Hemopexin	Q91 × 72	Hpx	−2.4
Non-protein transport (transport of iron)	Serotransferrin	Q921I1	Tf	−2.0
Non-protein transport (transport of lipids)	Apolipoprotein A-I	Q00623	Apoa1	−12.7
Non-protein transport (transport of lipids)	Microsomal triglyceride transfer protein large subunit	O08601	Mttp	−2.5
Other	Cullin-associated NEDD8-dissociated protein 1	Q6ZQ38	Cand1	−2.6
Other	Keratin, type I cytoskeletal 19	P19001	Krt19	−3.0
Other	Keratin, type II cytoskeletal 5	Q922U2	Krt5	−2.5
Other	Leucine-rich PPR motif-containing protein, mitochondrial	Q6PB66	Lrpprc	−3.0
Other	Protein FAM135A	Q6NS59	Fam135a	−3.2
Protein translation factor	Eukaryotic translation initiation factor 4 gamma 1	Q6NZJ6	Eif4g1	−4.6
Protein translation factor	**Elongation factor 1α-1**	P10126	Eef1a1	−3.3
Protein transport (import to nucleus)	Importin subunit β1	P70168	Kpnb1	−5.6
Protein transport (import to nucleus)	Importin-5	Q8BKC5	Ipo5	−2.5
Protein transport (sorting in the Golgi)	AP-1 complex subunit β1	O35643	Ap1b1	−2.1
Protein transport (sorting in the Golgi)	AP-1 complex subunit γ1	P22892	Ap1g1	−2.9
Protein transport (vesicle coat protein)	Clathrin heavy chain 1	Q68FD5	Cltc	−5.8
Protein transport (vesicle coat protein)	Coatomer subunit β1	Q9JIF7	Copb1	−6.9
Protein transport (vesicle-mediated transport)	AP-3 complex subunit Δ1	O54774	Ap3d1	−18.2
Protein transport between vesicular structures and Golgi	Vacuolar protein sorting-associated protein 35	Q9EQH3	Vps35	−4.9
Transcriptional regulation	HMyb-binding protein 1A	Q7TPV4	Mybbp1a	−2.1

Fold changes of up-regulated and down-regulated proteins are shown in red and blue, respectively. The underlined bold means that the protein was differentially expressed in both colon and ileum. * Negative fold change value means that the protein abundance was decreased by 5-FU treatment.

**Table 3:** Complexes of TFs potentially involved in differential gene expression in the gut of 5-FU treated mice.

Changes in Gene and Protein Expression	TRANSFAC Site Model ID	Potential Complex	Gene Symbol
Up-regulated in the colon	V$BCL6_Q3_01	Complex **1**	BCL6
	V$CETS1P54_02		ETS1
	V$NANOG_01		NANOG
	V$NR3C1_13		NR3C1
	V$DATF1_01	Complex **2**	DIDO1
	V$MYOGENIN_Q6		MYOG
	V$POU2F1_04		POU2F1
	V$SOX10_07		SOX10
Down-regulated in the colon	V$CRX_Q6	Complex **1**	OTX1, OTX2, CRX
	V$E2A_Q2		MYOD1, MYOG, MYF6, TCF3
	V$NF1C_02		NFIC
	V$STAT_Q6		STAT1, STAT2, STAT3, STAT4, STAT5A, STAT5B, STAT6
	V$EMX2_05	Complex **2**	EMX2
	V$FOSL2_02		FOSL2
	V$NF1C_Q6		NFIC
	V$TAL1_Q6		TAL1
	V$TFAP4_06		TFAP4
Up-regulated in the ileum	V$FOXM1_05	Complex **1**	FOXM1
	V$IK_Q5_01		IKZF1
	V$OTX2_Q3		OTX2
	V$P53_03		TP53
	V$ERBETA_Q5		ESR2
	V$ERBETA_Q5	Complex **2**	ESR2
	V$CP2_01		TFCP2
	V$ETV5FOXO1_02		ETV5, FOXO1
	V$STAT6_05		STAT6
	V$TFAP4_02		TFAP4
Down-regulated in the ileum	V$FOXO3_03	Complex **1**	FOXO3
	V$GKLF_Q3		KLF4
	V$NURR1_Q3		NR4A2
	V$RORA_09		RORA
	V$AR_12	Complex **2**	AR
	V$CEBPB_06		CEBPB
	V$IK1_01		IKZF1
	V$MYOGENIN_Q6		MYOG
	V$SOX2_Q4		SOX2

**Table 4:** Master regulators of genes differentially expressed in the gut of 5-FU-treated mice.

Protein	UniProt ID	Gene Symbol	Functional Group	Colon	Ileum
Serum albumin	P07724	*Alb*	Non-protein transport		Down
Apolipoprotein A-I	Q00623	*Apoa1*	Non-protein transport (transport of lipids)		Down
Cadherin-1	P09803	*Cdh1*	Cytoskeleton and cell adhesion (cell–cell adhesions)		Up
Chymotrypsin-like elastase family member 2A	P05208	*Cela2a*	Proteolysis (digestive enzyme)		Up
Calcium-activated chloride channel regulator 1	Q9D7Z6	*Clca1*	Mucus (regulation of mucus production)	Down	
Catenin α-1	P26231	*Ctnna1*	Cytoskeleton and cell adhesion (cell–cell adhesions)		Down
Dedicator of cytokinesis protein 2	Q8C3J5	*Dock2*	Cytoskeleton and cell adhesion (cytoskeleton regulation)		Up
Epithelial cell adhesion molecule	Q99JW5	*Epcam*	Cytoskeleton and cell adhesion (cell–cell adhesions)		Down
Protein Fga	E9PV24	*Fga*	Immune system (blood coagulation, innate and adaptive immunity)	Up	Down
Glutamine-fructose-6-phosphate aminotransferase [isomerizing] 1	P47856	*Gfpt1*	Metabolism	Down	Down
Glutathione peroxidase 1	P11352	*Gpx1*	Metabolism		Up
High mobility group protein B1	P63158	*Hmgb1*	Immune system		Up
Hemopexin	Q91 × 72	*Hpx*	Non-protein transport (transport of iron)		Down
Heat shock protein HSP 90-β	P11499	*Hsp90ab1*	Chaperone		Down
HMyb-binding protein 1A	Q7TPV4	*Mybbp1a*	Transcriptional regulation		Down
Nucleolin OS	P09405	*Ncl*	Other	Up	
Phosphatidylethanolamine-binding protein 1	P70296	*Pebp1*	Proteolysis (protease inhibitor)		Up
Periostin	Q62009	*Postn*	Cytoskeleton and cell adhesion (cell adhesion)	Down	
Protein Pzp	Q61838	*Pzp*	Proteolysis (protease inhibitor)	Up	
Superoxide dismutase [Cu-Zn]	P08228	*Sod1*	Antioxidant		Down
Serotransferrin	Q921I1	*Tf*	Non-protein transport (transport of iron)		Down
Talin-1	P26039	*Tln1*	Cytoskeleton and cell adhesion (cytoskeleton-membrane connection and cell–cell adhesion)		Down

‘Up’ and ‘Down’ mean that the master regulator is potentially responsible for up- or down-regulation of genes, given that the master regulator itself is up- or down-regulated.

**Table 5:** Potential 5-FU targets, a.k.a. master regulators of gene and protein expression changes, in the mouse gut.

Gene/Protein Changes	Pa	Pi	Predicted 5-FU Target/MOA	Uniprot ID	Gene Symbol
Up-regulated in the colon	0.410	0.079	Insulin-like growth factor 1 receptor/agonist	P08069	*IGF1R*
Down-regulated in the colon	0.500	0.034	Ca^2+^/calmodulin-dependent protein kinase A/inhibition	Q8N5S9	*CAMKK1*
	0.496	0.113	14-3-3γ/inhibition	P61981	*YWHAG*
	0.474	0.070	EphB2 kinase/inhibition	P29323	*EPHB2*
	0.456	0.105	Transcription factor STAT1/inhibition	P42224	*STAT1*
	0.363	0.083	IRS1/inhibition	P35568	*IRS1*
Up-regulated in the ileum	0.410	0.079	Insulin-like growth factor 1 receptor/agonist	P08069	*IGF1R*
Down-regulated in the ileum	0.500	0.034	Ca^2+^/calmodulin-dependent protein kinase A/inhibition	Q8N5S9	*CAMKK1*
	0.496	0.113	14-3-3γ/inhibition	P61981	*YWHAG*
	0.485	0.086	S-100B/inhibition	P04271	*S100B*
	0.474	0.070	EphB2 kinase/inhibition	P29323	*EPHB2*
	0.363	0.083	IRS1/inhibition	P35568	*IRS1*

Values Pa (activation) and Pi (inhibition) are the probabilities of the respective MOA. The terms were grouped according to the changes in protein levels (up- or down-regulation in the colon or ileum). MOAs are ordered from the top downwards by descending Pa values.

## Data Availability

Data are contained within the article.
